# Isolated bone marrow gamma/delta T-cell lymphoma: A difficult case to classify according to the current WHO classification of lymphoid malignancies

**DOI:** 10.1016/j.lrr.2025.100539

**Published:** 2025-08-08

**Authors:** Hideto Hyuuga, Naoki Oishi, Takuma Kumagai, Minori Matuura, Ayato Nakadate, Yuma Sakamoto, Jun Suzuki, Megumi Suzuki, Megumi Koshiisi, Ichiro Kawashima, Takeo Yamamoto, Kei Nakajima, Masaru Tanaka, Tetuso Kondo, Keita Kirito

**Affiliations:** a Department of Hematology and Oncology, University of Yamanashi, Chuo-shi, Yamanashi, Japan; b Department of Pathology, University of Yamanashi, Chuo-shi, Yamanashi, Japan

**Keywords:** T-cell lymphoma, Gamma/delta, Bone marrow

Dear editor

Gamma-delta T-cell lymphoma (GDTCL) is an extremely rare subgroup of mature T-cell lymphoma[1]. In general, GDTCL can present as hepatosplenic T-cell lymphoma (HSTCL), primary cutaneous T-cell lymphoma (PCTCL), monomorphic epitheliotropic intestinal T-cell lymphoma (MEITL) or gamma-delta large granular lymphocytic leukemia, as described in the current World Health Organization (WHO) classifications[2] or the international classification of mature lymphoid neoplasms [3]. Interestingly, Singh and colleagues have recently reported a case of GDTCL with isolated bone marrow invasion, that is difficult to classify under the current classification system in this journal. Based on these findings, they emphasized the necessitates for proper classification to cover these cases, especially for the patient with isolated bone marrow involvement [4]. We have read the report with great interest.

Here, we present the case of a patient with GDTCL who initially presented with pancytopenia and diagnosed with isolated bone marrow invasion of malignant cells. Our experience supports the proposal of Singh and colleagues.

## Case presentation

A 74-year-old male patient was referred to our hospital due to progressive pancytopenia with general fatigue and shortness of breath, starting within one month.

A peripheral blood test revealed severe anemia (Hb 6.5 g/dL), thrombocytopenia (platelet number; 2.8 × 10^9^/L) and neutropenia (neutrophil 1.0 × 10^9^/L). An increase in large lymphocytes was not found in the peripheral blood. Bone marrow biopsy revealed diffuse infiltration of abnormal medium-sized lymphoid cells with reticulin fibrosis (Fig.1A). Immunohistochemistry revealed that the neoplastic cells were positive for CD7, CD56 and T -cell receptor (TCR)δ, but negative for CD117, TCRβ, CD3, CD5, terminal deoxynucleotidyl transferase (TdT), CD34, CD4, CD8, CD30, granzyme B (Fig. 1A) and TIA1 (data not shown). *In situ* hybridization for EBER was negative (data not shown). Flow cytometry analysis of bone marrow aspirates confirmed that the neoplastic cells expressed only CD7 and CD56 (Fig. 1B). Monoclonal rearrangement of the TCR gamma gene (*TRG*) was detected by PCR, while no *JAK2* V617F, *CALR (Calreticulin)* exon 9 or *MPL* mutations were detected. Conventional cytogenetic culture resulted in failure. These results indicated that the patient had mature T-cell lymphoma expressing gamma/delta T-cell receptors. However, the patient did not have any cutaneous lesions, and ^18^F-fluorodeoxyglucose positron emission tomography (^18^F-FDG-PET/CT) revealed the accumulation of FDG only in the bone marrow but not in the liver, spleen, gastrointestinal tract or peripheral lymph nodes. In addition, neither hepatomegaly nor splenomegaly were found. Overall, we diagnosed the patient with isolated bone marrow GDTCL, as recently reported by Singh R and colleagues [4]. The patient was treated with a reduced dose regimen consisting of cyclophosphamide, doxorubicin, vincristine, and prednisolone. After 6 treatment cycles, his pancytopenia was ameliorated, and ^18^F-FDG-PET/CT indicated the resolution of abnormal accumulation in the bone marrow. Furthermore, bone marrow biopsy revealed the disappearance of abnormal lymphoid cells and reduction of reticulin fibers.

Physiologically, T cells with gamma/delta-TCRs are predominantly located in the red pulp of the spleen, epithelium of the gastrointestinal tract and skin[5]. Therefore, most GDTCLs, such as HSTCLs or PCTCLs, arise in the liver, spleen, and skin[1]. Although bone marrow is one of the common lesions that gamma-delta T cell lymphoma cells invaded in HSTCL in late phase of the disease, GDTCL cases primarily evolve from the bone marrow without hepatosplenic or skin manifestations were quite rare [4,6,7]. Sindhu et.al. reported a 74-year-old female case with involvement of malignant cells with gamma-delta T cell lymphoma phenotype both in peripheral blood and bone marrow without hepatosplenomegaly and skin lesions [6] as initial presentation. Turotte et.al. presented a 63 year old female case who initially presented with progressive pancytopenia [7]. Bone marrow biopsy revealed the invasion of malignant cells with features of CD8-positive gamma-delta T cell. CT imaging did not show evidence of nodal or extra nodal involvement of lymphoma [7]. Case series study of GDTCLs from Australian multicenter registry data included one GDTCL patient with nodal and bone marrow involvement who did not fit any type of WHO-defined GDTCL[8]. Very recently, Singh and colleagues reported a 59-year-old male case of isolated bone marrow gamma-delta T cell lymphoma [4]. In accord with these cases, our present case initially presented with anemia and thrombocytopenia and bone marrow biopsy revealed the invasion of malignant gamma-delta T cells, whereases the patient did not have hepatosplenomegaly, cutaneous abnormality, and gastrointestinal involvement at that time. Therefore, we diagnosed the case as an isolated bone marrow gamma-delta T cell lymphoma as proposed by Singh and colleagues [4]. It is important to distinguish bone marrow derived GDTCL from immature gamma-delta T cell acute leukemia /lymphoma (T-ALL). Usually, the neoplastic cells of gamma-delta T cell acute leukemia /lymphoma express markers of immature phenotype including CD34, CD1a or TdT [9,10]. As described above, the immunohistochemical study of bone marrow of our patient revealed that the malignant cells were negative for these immature cell markers in the present case. These notions indicated that the malignant cells of this patient had mature phenotype and support the diagnosis of bone marrow derived GDTCL, not immature gamma-delta T-ALL. Fig. 1a,b

**Fig.1A:**
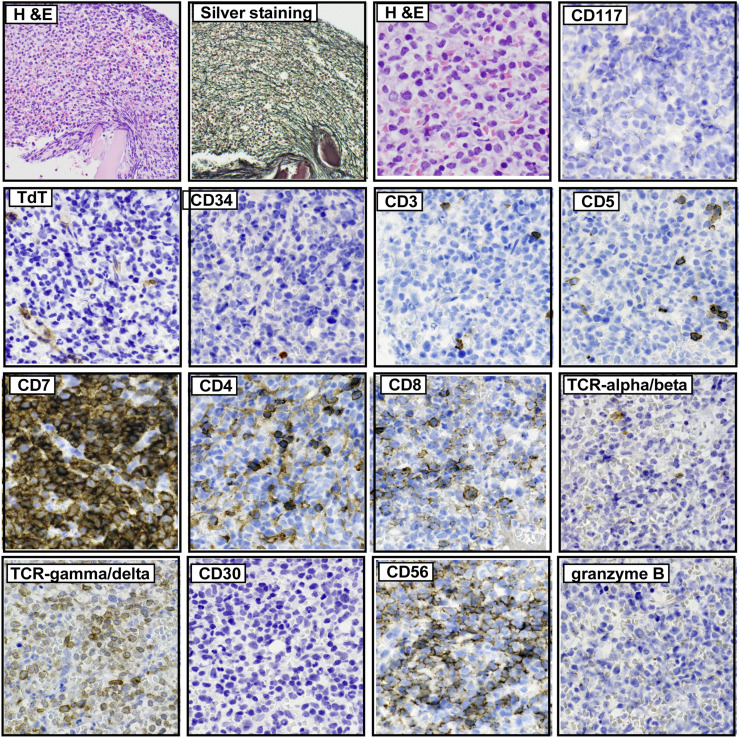
Histopathology of the trephine biopsy of bone marrow. The biopsy specimens were visualized with hematoxylin and eosin (H&E) staining or silver staining. Immunohistochemical analysis with the indicated antibodies was also performed.

**Fig.1B:**
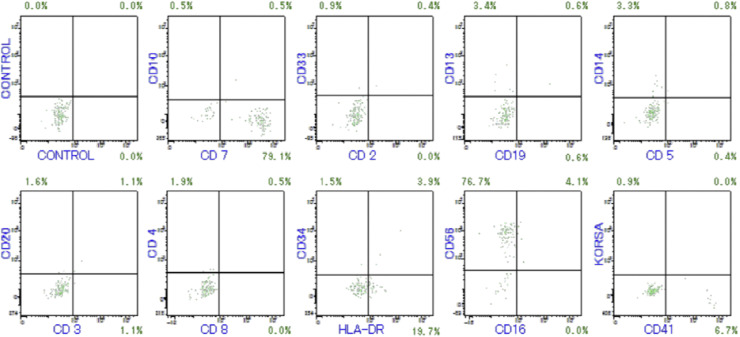
Flow cytometry of bone marrow aspirates.

In conclusion, we presented a case of GDTCL with isolated bone marrow invasion. It has been emphasized that these cases are difficult to classify according to the current WHO classifications [4,6] and that it is necessary for the new classification system to include cases of isolated bone marrow GDTCL without hepatosplenic or cutaneous involvement. The case presented here also supports these notions.

**Ethics approval**: All procedures performed were in accordance with the ethical standards of the institutional and national research committees and with the 1964 Helsinki Declaration and its later amendments or comparable ethical standards.

**Consent to participate**: Informed consent was obtained from the patients to publish information related to their clinical data.

**COI disclosure**: Keita Kirito; Honoraria Pharmaessentia Japan

**Funding Declaration:** There was no funding.

## CRediT authorship contribution statement

**Hideto Hyuuga:** Data curation. **Naoki Oishi:** Methodology, Data curation. **Takuma Kumagai:** Data curation. **Minori Matuura:** Data curation. **Ayato Nakadate:** Data curation. **Yuma Sakamoto:** Data curation. **Jun Suzuki:** Data curation. **Megumi Suzuki:** Data curation. **Megumi Koshiisi:** Data curation. **Ichiro Kawashima:** Data curation. **Takeo Yamamoto:** Data curation. **Kei Nakajima:** Data curation. **Masaru Tanaka:** Data curation. **Tetuso Kondo:** Writing – review & editing. **Keita Kirito:** Writing – original draft, Conceptualization.

## Declaration of competing interest

The authors declare that they have no known competing financial interests or personal relationships that could have appeared to influence the work reported in this paper.
